# Volume reduction of caudate nucleus is associated with movement coordination deficits in patients with hippocampal atrophy due to perinatal hypoxia-ischaemia

**DOI:** 10.1016/j.nicl.2020.102429

**Published:** 2020-09-15

**Authors:** Sharon Geva, Sebastian Jentschke, Georgios P.D. Argyropoulos, W.K. Chong, David G. Gadian, Faraneh Vargha-Khadem

**Affiliations:** a Cognitive Neuroscience and Neuropsychiatry Section, UCL Great Ormond Street Institute of Child Health, 30 Guilford Street, London WC1N 1EH, United Kingdom; b Developmental Imaging and Biophysics Section, UCL Great Ormond Street Institute of Child Health, London WC1N 1EH, United Kingdom

**Keywords:** Hypoxia-ischaemia, Motor control, Voxel-based morphometry, Caudate nucleus, Thalamus

## Abstract

Acute sentinel hypoxia-ischaemia in neonates can target the hippocampus, mammillary bodies, thalamus, and the basal ganglia. Our previous work with paediatric patients with a history of hypoxia-ischaemia has revealed hippocampal and diencephalic damage that impacts cognitive memory. However, the structural and functional status of other brain regions vulnerable to hypoxia-ischaemia, such as the basal ganglia, has not been investigated in these patients. Furthermore, it is not known whether there are any behavioural sequelae of such damage, especially in patients with no diagnosis of neurological disorder. Based on the established role of the basal ganglia and the thalamus in movement coordination, we studied manual motor function in 20 participants exposed to neonatal hypoxia-ischaemia, and a group of 17 healthy controls of comparable age. The patients’ handwriting speed and accuracy was within the normal range (Detailed Assessment of Speed of Handwriting), and their movement adaptation learning (Rotary Pursuit task) was comparable to the control group’s performance. However, as a group, patients showed an impairment in the Grooved Pegboard task and a trend for impairment in speed of movement while performing the Rotary Pursuit task, suggesting that some patients have subtle deficits in fine, complex hand movements. Voxel-based morphometry and volumetry showed bilateral reduction in grey matter volume of the thalamus and caudate nucleus. Reduced volumes in the caudate nucleus correlated across patients with performance on the Grooved Pegboard task. In summary, the fine movement coordination deficit affecting the hand and the wrist in patients exposed to early hypoxic-ischaemic brain injury may be related to reduced volumes of the caudate nucleus, and consistent with anecdotal parental reports of clumsiness and coordination difficulties in this cohort.

## Highlights

• We studied manual function in patients exposed to neonatal hypoxia-ischaemia.
• Patients had preserved function in motor adaptation learning, and in speed and accuracy of handwriting.
• Manual coordination was impaired on a timed pegboard task.
• Motor impairment was associated with bilaterally reduced volumes of the caudate nuclei.
• Early basal ganglia damage may lead to persistent deficits in fine manual control.

## 1 Introduction

There is strong evidence indicating that a consequence of acute sentinel hypoxic-ischaemic (HI) events experienced early in life is damage to specific subcortical brain structures, namely, the hippocampus which underpins cognitive memory and learning, the basal ganglia, which support habit memory and skill learning, as well as the thalamus (Caine and Watson, 2000, de Vries and Groenendaal, 2010, Dzieciol et al., 2017, Faro and Windle, 1969, Gadian et al., 2000, Guderian et al., 2015, Sie et al., 2000).

Our previous reports documented the association between hippocampal atrophy following early HI and memory impairment (Cooper et al., 2015, Gadian et al., 2000, Guderian et al., 2015, Muñoz-López et al., 2017, Vargha-Khadem et al., 2003, Vargha-Khadem et al., 1997). Furthermore, our group and others have highlighted that early HI can also affect structures which are functionally connected to the hippocampus, thus compromising the wider memory network, such as the mammillary bodies and anterior thalamus (Dzieciol et al., 2017, Guderian et al., 2015, Molavi et al., 2019).

HI injury targeting the basal ganglia and its circuitry has been mostly associated with overt motor deficits, as in cerebral palsy (Brown et al., 1974, Perlman, 1997, Volpe, 2008). Even in patients without overt motor deficits, studies have documented atrophy of basal ganglia structures (Guderian et al., 2015) as well as in the thalamus (Dzieciol et al., 2017). However, less is known about the behavioural consequences of such occult damage in this population. Regions within the basal ganglia and the thalamus form part of the brain’s motor control circuits (Alexander et al., 1991, McFarland and Haber, 2000) and have well-established roles in motor coordination (Dudman and Krakauer, 2016, Galvan et al., 2016, Jin et al., 2014, Stephenson-Jones et al., 2011). This is consistent with anecdotal parental reports indicating that children who had suffered from early HI, later show clumsiness and incoordination even in the absence of diagnosed neuromotor deficits (Gadian et al., 2000).

Here, we assessed hand motor coordination skills in relation to their neural correlates in individuals with a history of perinatal HI and evidence of hippocampal atrophy, but without a clinical diagnosis of motor impairment. The clinical presentation of the patients at birth and their abnormally small hippocampal volumes, were treated as markers of HI injury. We hypothesised that: (a) the patient group would show impairments lasting into late childhood and early adulthood in tasks requiring the execution of complex manual actions and sequences; and, (b) basal ganglia and thalamic volumes would predict scores in motor tasks where patients show impaired performance as compared to controls.

## 2 Materials and methods

### 2.1 Participants

Twenty patients with hippocampal volume reduction caused by exposure to HI events during the perinatal period were selected for the study (age at test: 14.05 ± 3.86 years [mean ± std. dev.], range: 9 – 25; 14 males). Hippocampal atrophy and documented perinatal semiologies were treated as markers of exposure to the HI events. Hippocampal volumes were measured (DGG) in each hemisphere, corrected for total intra-cranial volume (ICV) and averaged across hemispheres. Volumes were compared to the mean of an independent group of healthy controls (Cooper et al., 2015). The patient group had an average hippocampal volume of 2264 ± 503 mm^3^, whereas the control group had an average hippocampal volume of 3288 ± 229 mm^3^. Nine of the participants were selected from a cohort of patients diagnosed with Acute Hypoxemic Respiratory Failure (AHRF) who subsequently received Extracorporeal Membrane Oxygenation (ECMO; Field et al., 1996). The most common aetiologies were persistent pulmonary hypertension of the newborn due to meconium aspiration, congenital diaphragmatic hernia, isolated persistent foetal circulation, sepsis, and idiopathic respiratory distress syndrome. An additional 8 participants were selected from a cohort of patients who underwent a corrective Arterial Switch Operation (ASO) for dextro-transposition of the great arteries (TGA; Muñoz-López et al., 2017). Patients included were diagnosed as neurologically normal based on a detailed screening questionnaire. Table 1 provides details of aetiology, neuroradiological ratings, and degree of hippocampal volume reduction of each patient (see Table 1).

**Table 1:** Aetiology, neuroradiological ratings of T1-weighted scans, and mean % hippocampal volume reduction of patients.

Code	Aetiology	Neuroradiological Ratings						Hipp. atrophy (%)
		Hippo-campus	Fornix	MB	CC	PVWM	Other	
P1	Cyanosis at birth, ventilation, Postnatal TGA diagnosis & surgery	small	small	small	N	N		−47
P2	Severe AHRF & hypotonia at birth, pulmonary distress at 3 weeks, congenital hypothyroidism	small	very small	small	N	N		−52
P3	Birth asphyxia, severe AHRF	small	very small	small	G > S	N	focal abnormality L claustrum	−57
P4	Cyanosis at birth, postnatal TGA diagnosis, cardiac failure post-surgery	small	small	small	N	R focal abnormality		−53
P5	Premature (35 wks) AHRF, Cardiac & respiratory arrest; resuscitation, ventilation	small	small	small	N	N		−58
P6	AHRF & ECMO	N	small	small	N	N		−27
P7	AHRF & ECMO	suspected small bodies	N	N	G > S	N		−23
P8	AHRF & ECMO	N	N	small	N	N		−22
P9	AHRF & ECMO	N	N	N	N	N		−19
P10	AHRF & ECMO	small bodies	small	small	G > S	N		−19
P11	AHRF & ECMO	small	small	small	G > S	N		−32
P12	AHRF & ECMO	small	small	small	G > S	N		−50
P13	AHRF & ECMO	N	N	N	N	N		−16
P14	AHRF & ECMO	N	N	N	N	N		−20
P15	TGA	N	N	N	N	N		−20
P16	TGA	–	–	–	–	–	–	−22
P17	TGA	N	N	N	N	N		−22
P18	TGA	N	N	N	G > S	N	mild plagiocephaly	−17
P19	TGA	small	small	N	G > S	N		−17
P20	TGA	N	N	N	G > S	N	R poly-microgyria	−21

AHRF = Acute Hypoxemic Respiratory Failure; CC = corpus callosum; ECMO = Extracorporeal Membrane Oxygenation; G > S = genu > splenium; L = left; MB = mammillary bodies; N = normal; PVWM = peri-ventricular white matter; R = right; TGA = Transposition of the Great Arteries; % = percent reduction in mean hippocampal volume (see Section 2.1).

The patient group had a full-scale IQ in the normal range with a standard score mean of 100 ± 16 (using the Wechsler Intelligence Scale for Children-IV; Wechsler, 2003, or the Wechsler Adult Intelligence Scale; Wechsler, 1955). Attentional abilities were also in the normal range with scaled scores of 8.8 ± 3.5 for sustained attention, and 9.2 ± 3.2 for selective attention (using the Test of Everyday Attention – Children; Manly et al., 1999; or the Test of Everyday Attention; Robertson et al., 1994). By contrast, the patient group was impaired in overall memory function with a standard score in the General Memory scale of 80 ± 19 (using the Children’s Memory Scale; Cohen, 1997; or the Wechsler Memory Scale; Wechsler, 2009).

Seventeen healthy volunteers (age: 16.24 ± 7.05 years, range: 9 – 35; 9 males) served as controls. Of the 20 patients, 15 were right-handed, three were ambidextrous, and two were left-handed. Of the 17 control participants, 11 were right-handed, three were ambidextrous, and three were left-handed. Patients and healthy controls did not differ in age (Mann-Whitney *U* test, p = 0.66), sex, or handedness (χ^2^ tests for both, *p* > 0.24). Participants had no genetic syndromes and all were native English speakers. The study was approved by the London-Bentham Research Ethics Committee (ref. 05/Q0502/88) and all participants gave written informed consent, or assent and parental consent, according to the Declaration of Helsinki, before participating in the study.

### 2.2 Behavioural assessment

Participants completed the following four tasks requiring hand motor coordination: (1) **Grooved Pegboard** – a standardised test assessing fine motor coordination of the wrist and fingers.^5^ In this test, participants insert with the dominant or the non-dominant hand pegs into holes with different orientations. The task is administered twice using each hand, and the time required to insert all the pegs is recorded in seconds. (2) **Rotary Pursuit** (Ammons, 1995) – a task assessing movement adaptation of the hand and arm under time constraint. Participants track a moving light, by keeping contact between the moving light and a hand-held wand. The light moves in a circular trajectory, at a constant pace and in a clockwise direction. Initially participants practised the task three times: each trial lasted for 20 s, and the light was moving at a speed of 30 rounds per minute. Then a maximum of five trials was used to determine the baseline speed for each participant. The baseline speed is the fastest speed at which the participant kept the wand on the target for 25% of the time (i.e. time-on-target being 4.5 – 5.5 s, during a 20 s trial). The task was then administered in 4 blocks, each containing 8 trials of 20 s each, interspersed by 20 s of rest. Main outcome measure was the time on target, averaged across the 8 trials of each block. Three blocks were administered consecutively, and the fourth block was administered the next day to measure adaptation learning after overnight delay. Additional variables assessed included: (a) Baseline speed: Speed required for the participant to stay on target for 25% of the time; (b) Learning parameter: [average time-on-target on block 4] minus [average time-on-target on block 1]. (3) **Detailed Assessment of Speed of Handwriting** (DASH; Barnett et al., 2007) – a standardised test assessing speed of handwriting in three subtests: (a) neat sentence copying; (b) fast sentence copying; and (c) fast alphabet writing. (4) **Beery-Buktenica Developmental Test of Visual Motor Integration** (VMI; Beery and Beery, 2004) – a standardised test assessing (a) visual-motor integration (copying shapes), (b) visual perception (visual comparison task) and (c) motor coordination (drawing between the lines of geometric shapes with time for completion recorded).

### 2.3 MRI data acquisition and assessment

MRI scans were obtained using a 1.5T Siemens Avanto (Germany) MRI scanner. T1-weighted 3D Fast Low Angle Shot (FLASH) scans (repetition time (TR) = 11 ms, echo time (TE) = 4.94 ms, flip angle = 15°, field of view = 224 × 256 mm, 176 slices, sagittal plane, voxel size: 1 × 1 × 1 mm) were used for the neuroradiological examination of the scans.

A consultant paediatric neuroradiologist (WKC) provided radiological rating**s** of structural brain abnormalities by visually inspecting all healthy participants’ and 19 / 20 patients’ T1-weighted MRI scans, while being blinded to participants’ diagnosis and group affiliation. The following pre-defined brain structures were rated as either ‘normal’ or ‘small’: hippocampus, extra-hippocampal structures within the medial-temporal lobe, fornix, dorsomedial thalamus, mammillary bodies, basal ganglia, and cerebellum. The size of the mammillary bodies was compared to internal landmarks: optic chiasm or the anterior commissure. The lateral ventricle was rated for being ‘normal’ or ‘enlarged / dilated’. Periventricular white matter was rated for amount (‘normal’ or ‘reduced’). The sizes of the genu and the splenium of the corpus callosum were compared, where splenium > genu was rated as ‘normal’; while a reversed relationship (genu > splenium) was rated as ‘abnormal’. Other abnormalities such as ischaemic or atrophic damage, haematoma or gliotic scars were noted as well. The basal ganglia were reported as ‘normal’ in all patients, together with the extra-hippocampal structures within the medial temporal lobe, thalamus, lateral ventricle and cerebellum. No detectible abnormalities were reported in the controls. See Table 1 for neuroradiological findings.

### 2.4 Data analysis

#### 2.4.1 Behavioural data analysis

The rotary pursuit task was not performed by one patient and two control participants due to lack of time or technical difficulties administering the task. Other tasks were performed by all participants. Results for the non-dominant hand on the Grooved Pegboard task were excluded for one patient because they represented a very strong outlier (z = −46.1).

To assess whether there was a common factor underlying performance on the different motor tests, a Principal Component Analysis (PCA) was conducted on the behavioural data. This did not reveal any new relations between the variables: Four statistically reliable components were extracted (variables loading with a score > 0.4), together accounting for 76% of the variance. These four factors strongly corresponded to the four behavioural tests and therefore each of the behavioural tests was analysed separately.

Scores from the standardised tests (Grooved Pegboard, VMI, DASH) were analysed using parametric tests. We present scores converted to z-scores on all standardised tests for ease of interpretation. One-sample t-tests confirmed that our control group did not differ from the standard population in their performance (p > 0.3 for all). We therefore compared the patients’ performance to that of the control group using mixed-effects ANOVAs (2 groups: patients and controls, and a changing number of conditions; see Table 2), and followed these analyses with post-hoc t-tests. In addition, for each standardised test, we identified the number of patients who showed impaired performance (a score which was more than 1.5 standard deviations below the standard mean, in accordance with current UK educational guidelines). In the rotary pursuit task, average time on target was normally distributed (Shapiro-Wilk test, p > 0.05 for both controls and patients), while scores for the learning parameter and baseline speed were not normally distributed for controls and/or patients (Shapiro-Wilk test, p < 0.05). We used parametric tests (mixed-effects ANOVA and post-hoc *t*-test) for the former and non-parametric tests (Mann-Whitney U) for the latter.

#### 2.4.2 Voxel-based morphometry analysis

T1-weighted scans were processed using the Statistical Parametric Mapping software (SPM12 v7219, https://www.fil.ion.ucl.ac.uk/spm/) implemented in the Matlab environment (R2020a, The MathWorks Inc.; Natick, MA). Images were examined for scanner artefacts and were re-orientated so that the origin coordinates lay within the anterior commissure.

For the voxel-based morphometry (VBM) analysis, segmented whole-brain images were processed using the high-dimensional Diffeomorphic Anatomical Registration Through Exponentiated Lie algebra (DARTEL; Ashburner, 2007). This technique demonstrates higher normalization power by improving inter-subject alignment, especially for small inner structures (Yassa and Stark, 2009), and as such, is considered a better alternative to standard normalization (Ashburner and Friston, 2005). After affine registration of the grey matter (GM) DARTEL templates to the tissue probability maps in Montreal Neurological Institute (MNI) space, non-linear warping of GM images was performed to the DARTEL GM template in MNI space.

A modulation of segmented GM maps was undertaken to compensate for volume changes during the spatial normalization by multiplying the intensity value in each voxel with the Jacobian determinants. The total GM signal in the normalised images was thus preserved. Normalised modulated GM images were visually inspected for quality of the segmentation-normalisation process. Resulting image voxels measured 1.5 × 1.5 × 1.5 mm. Normalised modulated images were smoothed with an isotropic 8 mm full-width at half-maximum (FWHM) Gaussian kernel. The smoothed images were then analysed using a *t*-test contrast (Controls > Patients), including age, sex and ICV (Ashburner and Friston, 2005) as covariates, using whole-brain peak voxel-level family-wise error (FWE) correction (p < 0.05) over p < 0.001 (unc.). Given our a priori hypothesis on basal ganglia and thalamic structural abnormalities in the patient group, we report clusters confined within these regions in the DARTEL GM template in MNI space using small-volume correction, if whole brain-corrected level results are not found.

#### 2.4.3 Volumetry of subcortical structures

The whole-brain VBM analysis was followed up by automated segmentation of the thalamus and basal ganglia structures (caudate nucleus, putamen, nucleus accumbens, and globus pallidus), using FSL-FIRST v.6.0 (Patenaude et al., 2011) for all participants’ T1-weighted MRIs in native space. Volumes were segmented in each hemisphere, visually inspected for accuracy and corrected for ICV derived from the unified segmentation procedure (Ashburner and Friston, 2005) in SPM12. No difference was found between groups in ICV (t = 0.16, p = 0.871; ANCOVA, controlling for age: F_(1, 34)_ = 0.02, p = 0.901). Patients’ corrected volumes were compared against those of controls, and their relationship with age was investigated in a series of bivariate correlation analyses. The Holm-Bonferroni method was used to correct (‘p_corr’_) for multiple comparisons (Holm, 1979). As several volumetric measurements were not normally distributed (Shapiro-Wilk test: p < 0.05), the Mann-Whitney *U* test was employed for group comparisons. Greenhouse-Geisser correction of degrees of freedom was applied in our ANOVAs when the assumption of sphericity was violated (Mauchly’s test: p < 0.05).

#### 2.4.4 Structure-function relationships

We aimed to establish a relationship between manual coordination performance and structural integrity of the thalamus and basal ganglia structures. We thus entered patients’ volumes of the thalamus and basal ganglia structures in a series of bivariate correlation analyses along with indices of motor performance wherein patients showed impairment (SPSS, v.26). As several volumetric measurements and behavioural scores were not normally distributed (Shapiro-Wilk test, p < 0.05), non-parametric correlations (Spearman’s rho) were used.

## 3 Results

### 3.1 Behavioural performance

**Fig. 1:**
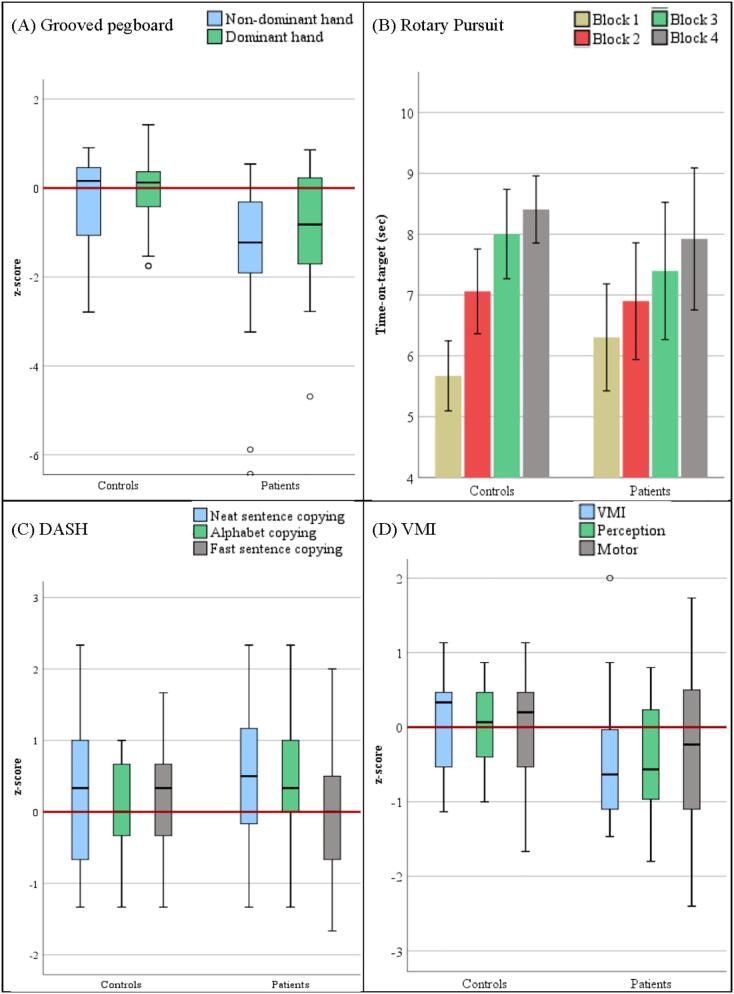
Participants’ behavioural scores (A) Grooved Pegboard; (B) Rotary Pursuit – average time-on-target in each block, bars represent ± 1 standard error; (C) DASH (transformed into z-scores), and; (D) VMI (transformed into z-scores). Red horizontal lines represent the population average. (For interpretation of the references to colour in this figure legend, the reader is referred to the web version of this article.)

The behavioural performance within each group is summarised in Table 2 and illustrated in Fig. 1.

**Table 2:** Performance scores on the manual coordination tests for the patient and the control groups.

Task	Controls		Patients		Controls vs. Patients	
	Mean (SD)	Impaired performance	Mean (SD)	Impaired performance	Test	p
*Grooved Pegboard*						
Dominant hand	−0.10 (0.94)	3 (18%)	−1.12 (1.65)	6 (32%)	F	0.018*
Non-dominant hand	−0.30 (1.01)	1 (6%)	−1.58 (1.86)	8 (42%)		

*Rotary Pursuit*						
Average time on target	4.82 (0.66)	N/A	4.97 (0.59)	N/A	F	0.787
Baseline speed(rounds / min)	50.20 (8.31)	N/A	43.63 (10.44)	N/A	U	0.060
Learning (Last – First block)	2.73 (1.06)	N/A	1.62 (2.46)	N/A	U	0.187

*DASH*						
Neat sentence copying	0.25 (1.01)	0 (0%)	0.53 (0.92)	0 (0%)	F	0.540
Fast sentence copying	0.18 (0.98)	0 (0%)	−0.03 (0.91)	2 (10%)		
Fast alphabet writing	0.06 (0.70)	0 (0%)	0.45 (0.85)	0 (0%)		

*VMI*						
Visual-motor integration	0.02 (0.73)	0 (0%)	−0.44 (0.89)	0 (0%)	F	0.085
Visual Perception	0.01 (0.55)	0 (0%)	−0.41 (0.74)	2 (10%)		
Motor	−0.01 (0.77)	1 (5.9%)	−0.32 (1.11)	4 (20%)		

z-scores are presented for the standardised tests (Grooved Pegboard, VMI, DASH). Impaired performance [number (percentage) of participants] is defined as z-score **<** −1.5. DASH – Detailed Assessment of Speed of Handwriting; VMI – Beery-Buktenica Developmental Test of Visual Motor Integration; SD – standard deviation; * p < 0.05. Note: Most measurements met the requirements for using parametric methods. When these requirements were met, Analyses of Variance were used for the group comparisons. This is indicated by ‘F’ in the column ‘Test’. If measurements deviated from a normal distribution, a non-parametric method was used instead (Mann-Whitney *U* test). Those are marked by ‘U’ in the column ‘Test’.

#### 3.1.1 Grooved pegboard

Patients performed worse than controls using both the dominant and the non-dominant hand. In addition, a large proportion of patients showed impaired performance, particularly with the non-dominant hand (see Table 2 and Fig. 1A). These differences were statistically analysed using a mixed-effects ANOVA with the factors Group (patient vs. control) and Hand (dominant vs. non-dominant). This revealed a significant main effect of Group (F_(1,34)_ = 6.16, p = 0.018) and a significant main effect of Hand (F_(1,34)_ = 5.14, p = 0.030), but their interaction was non-significant (F_(1,34)_ = 1.41, p = 0.244). Post-hoc evaluation using independent sample t-tests revealed that the group difference was significant for both hands (non-dominant hand: t = 2.51, p = 0.017; dominant hand: t = 2.26, p = 0.030). The number of participants with impaired performance significantly differed between groups for the non-dominant (χ^2^ = 6.28; p = 0.012), but not for the dominant hand (χ^2^ = 0.76; p = 0.383).

#### 3.1.2 Rotary pursuit

For the rotary pursuit, the two groups had similar performance in their average time on target, increasing over blocks (see Table 2 and Fig. 1B), although the controls generally showed a slightly better learning performance. However, when describing the learning performance as similar, it has to be considered that the speed with which the target was moving was about 12% higher in the control group than in the patient group (50 vs. 44 RPM), representing a trend towards a significant difference (U = 88.50, p = 0.060). Evaluating the time on target using a mixed-effects ANOVA with the factors Group (patient vs. control) and Blocks (4), revealed a significant linear trend of block (F_(2.19,70.09)_ = 25.25, p < 0.001), but no significant effects for group (F_(1,32)_ = 0.07, p = 0.787), or an interaction between the two factors (F_(2.19,70.09)_ = 2.19, p = 0.115). Results remained similar when statistically controlling for age and baseline speed (by using these variables as covariates). The two groups also did not differ in their motor learning ability (U = 104, p = 0.187).

#### 3.1.3 DASH

For the DASH, a general trend for better performance of one group versus the other was not found: Only a limited number of participants in the patient group showed performance that was classified as ‘impaired’ (see Table 2 and Fig. 1C). That patients were not impaired in their performance on the DASH was confirmed using a mixed-effects ANOVA with the factors Group (patient vs. control), and Subtest (neat sentence copying, fast alphabet writing, fast sentence copying). This analysis failed to reveal significant main effects of Group (F_(1,35)_ = 0.38, p = 0.540), or Subtest (F_(2,70)_ = 2.61, p = 0.081), or a significant interaction (F_(2,70)_ = 2.55, p = 0.086). In order to evaluate the influence of using 16-year old standardisation norms for older participants (see Section 2.2), we excluded the three patients and five control participants who were older than 16 years of age at the time of testing. As above, none of the effects were significant. Looking at individual patients, it was found that some patients showed impairment only on the fast sentence copying (see Table 2), but the number of participants with impaired performance on this task did not significantly differ between the two groups (χ^2^ = 1.79; p = 0.180).

#### 3.1.4 VMI

Patients’ performance was numerically lower on all three subtests of the VMI. In addition, for a higher proportion of the participants in the patient group, performance was categorised as impaired for the motor and perception tasks (see Table 2 and Fig. 1D). However, when the lower performance of the patients compared to the control group was evaluated using a mixed-effects ANOVA with the factors Group (patient vs. control) and Subtest (motor, perception, and integration), there were no significant effects for the Group factor (F_(1,35)_ = 3.15, p = 0.085), Subtest factor (F_(1.70,59.51)_ = 0.053, p = 0.926), nor the interaction (F_(1.70,59.51)_ = 0.17, p = 0.813). Similarly, the number of participants with impaired performance did not significantly differ between groups on any of the subtests (p ≥ 0.180).

### 3.2 Voxel-based Morphometry

**Fig. 2:**
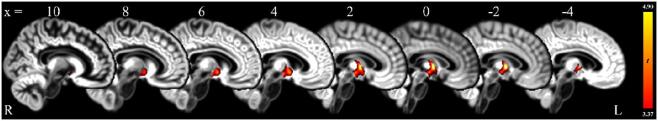
Clusters of reduced grey mater signal intensity in patients compared to controls, yielded by a whole-brain VBM analysis. Volume reduction in the anterior thalamus extending to the right caudate nucleus: voxels within this cluster survive peak-level FWE-correction (<0.05) for a mask comprising the thalamus and the basal ganglia (small volume correction; SVC) over p_unc_ < 0.001. Clusters are overlaid on a DARTEL GM template in MNI space (sagittal slices) and displayed over an individual voxel threshold of p_unc_ < 0.001; heat bar represents t-values; contrast: ‘controls > patients’.

Voxel-based Morphometry confirmed the patient group’s bilateral volume reduction in the hippocampus (Gadian et al., 2000), and additionally showed volume reduction in the anterior thalamus, extending to the right caudate nucleus (Fig. 2; Table 3).

**Table 3:** Whole-brain VBM of grey matter for contrast ‘controls > patients’

Correction level	Structure	Cluster size	Peak				
			p_FWE_	t	Coordinates (mm)		
		nvox			x	y	z
Whole brain	R Hippocampus	445	<0.001	7.73	22.5	−25.5	−16.5
	L Hippocampus	118	0.007	6.31	−26.0	−18.0	−20.0
Thalamus and basal ganglia (small volume correction)	Anterior thalamus extending to R caudate nucleus	320	0.011	4.90	−1.5	−3.0	3.0

nvox – number of voxels; R, L: right, left hemisphere.

### 3.3 Volumetry of subcortical structures

Consistent with the findings of our previous studies (Dzieciol et al., 2017, Gadian et al., 2000, Isaacs et al., 2003), patients showed bilateral volume reduction in the thalamus and the caudate nucleus (p_corr_ < 0.05 for all; Table 4).

**Table 4:** Automated volumetry of subcortical structures.

Structure	Controls		Patients		U	p_corr_	
	Median	IQR	Median	IQR			
Left Thalamus	5.59	0.40	5.29	0.42	64	0.009	**
Right Thalamus	5.46	0.55	5.13	0.53	73	0.023	**
Left Caudate Nucleus	2.75	0.34	2.46	0.24	78	0.032	**
Right Caudate Nucleus	2.81	0.43	2.44	0.27	82	0.049	**
Left Putamen	3.48	0.40	3.48	0.30	150	>0.999	
Right Putamen	3.38	0.27	3.31	0.31	140.5	>0.999	
Left Nucleus Accumbens	0.36	0.07	0.34	0.08	109.5	0.396	
Right Nucleus Accumbens	0.30	0.05	0.29	0.12	139	>0.999	
Left Pallidum	1.13	0.13	1.15	0.13	168	>0.999	
Right Pallidum	1.16	0.11	1.12	0.06	117	0.545	

Volumes are expressed as ‰ ICV; p_corr_: p values are adjusted for multiple comparisons using the Holm-Bonferroni procedure; IQR: inter-quartile interval; ** p_corr_ < 0.05.

### 3.4 Structure-function relationships

**Fig. 3:**
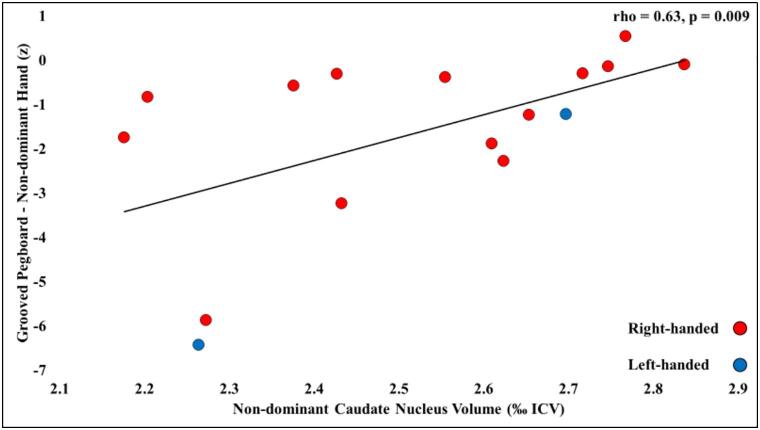
Correlation between non-dominant caudate nucleus volume and performance with the non-dominant hand in the Grooved Pegboard task across patients. Dependent measure: standardised scores (z) for performance on the Grooved Pegboard task with the non-dominant hand; volume is expressed as ‰ ICV; rho (ρ): Spearman’s correlation coefficient.

Patients’ reduced volume of the right caudate nucleus correlated with their impaired performance on the Grooved Pegboard task (rho = 0.45; p = 0.049; Fig. 3). This was driven by the correlation between this volume and right-handed patients’ performance using their non-dominant (left) hand (rho = 0.61, p = 0.020). A similar relationship was observed between performance scores for the non-dominant hand and the volume of the non-dominant caudate nucleus (not considering the 3 ambidextrous patients; rho = 0.63, p = 0.009). Such correlations were not observed with patients’ reduced left caudate or thalamic volumes and performance for either dominant or non-dominant hand (p > 0.21). Patients’ marginally lower baseline speed in the rotary pursuit task did not significantly correlate with the volume of any of their subcortical structures (left / right caudate nucleus, left / right thalamus: p > 0.304; Table 5).

**Table 5:** Structure-function relationships.

Structure	Grooved Pegboard		Rotary Pursuit Baseline speed	
	rho	p	rho	p
L Thalamus	0.10	0.682	0.25	0.304
R Thalamus	−0.16	0.502	0.13	0.598
L Caudate Nucleus	0.03	0.895	0.22	0.362
R Caudate Nucleus	0.45	0.049 *	−0.09	0.713

Bivariate correlations between volumes of structures that showed volume reduction and behavioural measures that showed significant / trending levels impairment across patients. Grooved pegboard: age-scaled standardised scores, averaged between the dominant and non-dominant hands; volume is expressed as ‰ ICV; rho: Spearmann’s rank correlation coefficient; *: p < 0.05. L: Left, R: Right.

Patients’ age did not correlate with their reduced left or right caudate nucleus volume or with their performance in the Grooved Pegboard task (all ps, p ≥ 0.672).

## 4 Discussion

In this study we investigated manual movement coordination in patients with a history of perinatal HI injury supported by clinical semiology and the documentation of bilaterally-reduced hippocampal volumes. Searching for behavioural correlates of potential basal ganglia abnormality, previous studies have examined the later motor development of children with a history of neonatal HI. It has been shown that during infancy and toddlerhood (up to the age of 4), a large number of children with a history of HI show gross motor abnormalities, when assessed with various standardised scales (e.g. Alberta Infant Motor Scale or the Bayley Scales of Infant Development, Gross Motor Function Classification System; de Vries and Jongmans, 2010, Steinman et al., 2009, van Schie et al., 2007, van Schie et al., 2010, Vermeulen et al., 2008). A study of 7-year-olds showed that as many as 20% of children with a history of HI have developmental dyspraxia (Hövels-Gürich et al., 2006). Some of these studies associated damage to the basal ganglia (and its circuity) to widely defined ‘poor outcome’ (de Vries and Jongmans, 2010, Vermeulen et al., 2008). Notably, these studies (1) examined general motor development without examining fine motor abilities, (2) studied only early childhood, and (3) have included the full range of motor problems from very mild to very severe (i.e. cerebral palsy). To address these caveats, we selected patients who had been exposed to HI events as neonates, and had confirmed bilateral hippocampal atrophy, but no overt evidence of atrophy in the basal ganglia, or deficits in motor function. We asked: (1) is there a behavioural impairment in fine motor movement in late childhood in this population? And, (2) if so, is it associated with occult damage to the thalamus and/or basal ganglia?

Our results indicate that a proportion of individuals in the patient group showed a significant deficit in complex manual movements, especially fine finger and wrist movements, and that the impairment was more pronounced in the non-dominant hand. These impairments are not uniform, as in each task, subgroups of individuals scored in the typical range. Importantly, we demonstrated for the first time an association between patients’ manual movement performance and the volume of the caudate nucleus. These structural differences may help explain some of the behavioural variation within the patient group.

We have shown that, as a group, patients were impaired on the Grooved Pegboard task (especially when using their non-dominant hand). By contrast, patients performed similarly to controls on speed of hand-writing (DASH), which involves a well-practiced movement that has been long-established by the time of this formal assessment. This suggests that the more automatic, and well-learnt motor skill of writing has developed to normal standards in our patient group. Moreover, in a task that is clinically used to dissociate between visual motor integration, motor coordination, and visual perception (VMI), we found only marginal, and non-selective differences between the two groups. Lastly, patients also showed motor adaptation learning (on the Rotary Pursuit task) similar to standards shown by the control group, despite a trend for a slower speed setting required for patients to complete the Rotary Pursuit task. Taken together, these findings suggest that some patients have difficulty in the performance of fine and novel manual movements, but normal ability to perform well-practiced writing tasks, and normal adaptive motor learning skill. Some parents reported that the children were clumsy during their early development, but most outgrew their motor incoordination, at least superficially, by the time they reached secondary school. However, here we have demonstrated that some patients have persistent deficits into adolescence and adulthood, even when there are no clinical signs of a motor deficit or a diagnosis of Developmental Coordination Disorder (American Psychiatric Association, 2013), and that this deficit can be detected when using novel and challenging manual tasks.

Patients’ non-dominant hand was more frequently affected than the dominant hand on the Grooved Pegboard task. This is in line with early findings showing that relative low proficiency of the non-dominant hand is associated with a neurological history (e.g. convulsions in infancy, meningitis, and head injury, as in Bishop, 1980). For the dominant hand, components of fine movement control have become automatised, since in real life it is the dominant hand that carries out the more finely-tuned movements. Motor performance using the non-dominant hand, on the other hand, may require visual guidance and cognitive control to a larger extent compared with that involving the dominant hand (Heuer, 2007, Studenka and Zelaznik, 2008). Consistent with the above, increased complexity of a novel finger tapping task was associated with larger hemodynamic response in the caudate nucleus and adjacent anterior putamen (Lehéricy et al., 2006).

We note that the handwriting task (DASH) only measures speed, but not quality or accuracy of handwriting. A previous study found that children with Developmental Coordination Disorder were impaired on the DASH, though a detailed analysis of their performance showed that the slowness was due to those children pausing frequently, rather than executing the movement more slowly (Prunty et al., 2013). Thus, even children with Developmental Coordination Disorder did not show impairment in the core motor component of the task. Future studies should measure quality of handwriting, as well as the speed in which the task is performed.

We further demonstrated that the impaired manual movement performance of patients is associated with reduced volume in the caudate nucleus. Caudate nucleus volume reduction in this population has been reported before (Guderian et al., 2015, Isaacs et al., 2003), but its functional significance had not been demonstrated. Previous studies highlight the significance of the caudate nucleus specifically in complex conditions of motor performance in healthy adults (Boisgontier et al., 2016). In particular, dopamine transporter availability in the neostriatum has been associated with performance on the grooved pegboard task in both healthy adults and patients with Parkinson’s disease as well as in methamphetamine abusers (Bohnen et al., 2007, Mozley et al., 2001, Volkow et al., 2001). Moreover, according to neurophysiological models of motor sequence learning, the circuits of the prefrontal and premotor cortices together with the ‘associative striatum’ (predominantly represented by the largest portion of the caudate nucleus as well as by the anterior putamen), support the implementation of sequential movements in the pre-automatic stage of learning. The automatic execution of motor sequences, on the other hand, relies on the circuits connecting the sensorimotor and parietal cortices with the ‘motor striatum’^6^ (Doyon et al., 2009, Penhune and Steele, 2012). This is in line with our findings that damage to the caudate nucleus in our patient group was associated with the performance of a novel motor task that requires precision, while a more practiced motor task such as writing shows unimpaired performance in our cohort of patients with neonatal exposure to HI. However, we also note that striatal damage has been associated with impaired learning in the rotary pursuit task in patients with Parkinson’s and Huntington’s disease (Gabrieli et al., 1997, Heindel et al., 1989, Heindel et al., 1988). Here, however, there were no significant learning differences between the control and the patient groups. While it might be the case that patients had an “easier” task at learning, given their marginally slower baseline speed, we also note that baseline speed was not predictive of learning performance. Several factors may account for this possible discrepancy. The neurodevelopmental nature of our patients’ condition may involve ample room for the development of compensatory mechanisms to support adequate levels of performance in motor skill learning. Other factors may pertain to the focality and severity of the striatal damage involved. Here, we observed subtle volume reduction in the caudate nucleus, but not in the putamen, consistent with our previous study of the larger cohort of patients exposed to early onset HI (Guderian et al., 2015). By contrast, Huntington’s and Parkinson’s diseases are associated with pronounced degeneration that extends beyond the caudate nucleus (Bäckman et al., 1997, Geng et al., 2006). This difference would be expected given that Huntington’s and Parkinson’s diseases are degenerative, whereas in the case of our cohort, the damage was caused by a single event. Moreover, other studies have suggested that medicated, non-demented patients with Parkinson’s disease in the early-mid stages of illness show relatively preserved learning on rotary pursuit paradigms (Bondi and Kaszniak, 1991, Heindel et al., 1989).

Consistent with our earlier findings (Dzieciol et al., 2017, Gadian et al., 2000, Isaacs et al., 2003), we showed that patients’ tissue volume and/or grey matter density reduction extended also to the thalamus. While there was no correlation between volume reduction in the thalamus and motor performance, it is noted that the thalamus is a complex structure with multiple connections to both cortical and subcortical regions, and only some of its parts are directly linked with motor function. Future studies which will delineate the thalamic sub-structures might be able to establish the association between damage to specific sub-structures and motor performance.

Additionally, the patient group is heterogeneous with regard to their aetiology, and hippocampal damage was used as a marker of the effects of the HI event. In our previous studies we did not find evidence of aetiological variables contributing to the reported pattern of hippocampal damage (Cooper et al., 2015, Vargha-Khadem et al., 2003). Future studies should evaluate whether the same conclusion can be drawn with regard to basal ganglia damage and its associated motor impairments, or whether different causes of HI have different effects on the basal ganglia damage described here.

In summary, we demonstrated that children who were exposed to episodes of HI in early life can continue to display deficits in fine, novel, and complex manual movement, in late childhood and early adulthood, even in the absence of overt neurological diagnosis. We also highlighted the significance of basal ganglia volume reduction in explaining the behavioural impairments observed. While it is known that the major sequel of exposure to HI is hippocampal damage, other regions besides the hippocampus may suffer damage as well. In this study we characterised the additional damage that may result from such injury, both behaviourally and structurally. This study brings us a step closer towards understanding the range of manual movement deficits that emerge with early exposure to HI. It is of interest in future research to examine whether the structure-function relation found here can be replicated in patients with pure ischaemic damage as well as those with pure hypoxic damage, each receiving brain protection through hypothermia. Notably, such patient groups are rare. Lastly, future studies employing other imaging techniques might shed more light on the neural foundations of manual motor impairments in patients with brain damage due to early HI.

## CRediT authorship contribution statement

**Sharon Geva:** Data curation, Formal analysis, Methodology, Visualization, Writing - original draft. **Sebastian Jentschke:** Conceptualization, Data curation, Formal analysis, Methodology, Writing - original draft. **Georgios P.D. Argyropoulos:** Formal analysis, Methodology, Visualization, Writing - original draft. **W.K. Kling Chong:** Development of methodology, Clinical review of MRI scans. **David G. Gadian:** Development of methodology. **Faraneh Vargha-Khadem:** Conceptualization, Data curation, Funding acquisition, Investigation, Methodology, Project administration, Resources, Supervision, Validation, Writing - review & editing.

## Declaration of Competing Interest

The authors declare that they have no known competing financial interests or personal relationships that could have appeared to influence the work reported in this paper.

## Footnotes

5 Standardisation is available only for ages 9 to 16. Scores of those participants who were older than 16 were based on the norms provided for 16-year-olds.

6 ‘Motor striatum’ refers to primarily posterior putamen, along with a smaller, lateral region in the caudate nucleus.
